# Semi-automated data collection from electronic health records in a stroke unit in Brazil

**DOI:** 10.1590/0004-282X-ANP-2020-0558

**Published:** 2021-12-07

**Authors:** Raquel Franco Zambom Valêncio, Juli Thomaz de Souza, Fernanda Cristina Winckler, Gabriel Pinheiro Modolo, Natalia Cristina Ferreira, Silmeia Garcia Zanati Bazan, Marcos Christiano Lange, Carlos Clayton Macedo de Freitas, Sergio Alberto Rupp de Paiva, Rogério Carvalho de Oliveira, Gustavo José Luvizutto, Rodrigo Bazan

**Affiliations:** 1 Universidade Estadual Paulista “Júlio de Mesquita Filho” Faculdade de Medicina de Botucatu Botucatu SP Brazil Universidade Estadual Paulista “Júlio de Mesquita Filho”, Faculdade de Medicina de Botucatu, Botucatu SP, Brazil.; 2 Universidade Estadual Paulista “Júlio de Mesquita Filho” Faculdade de Medicina de Botucatu Departamento de Medicina Interna Botucatu SP Brazil Universidade Estadual Paulista “Júlio de Mesquita Filho”, Faculdade de Medicina de Botucatu, Departamento de Medicina Interna, Botucatu SP, Brazil.; 3 Universidade Estadual Paulista “Júlio de Mesquita Filho” Faculdade de Medicina de Botucatu Departamento de Neurologia, Psicologia e Psiquiatria Botucatu SP Brazil Universidade Estadual Paulista “Júlio de Mesquita Filho”, Faculdade de Medicina de Botucatu, Departamento de Neurologia, Psicologia e Psiquiatria, Botucatu SP, Brazil.; 4 Universidade Federal do Paraná Complexo Hospital de Clínicas Curitiba PR Brazil Universidade Federal do Paraná, Complexo Hospital de Clínicas, Curitiba PR, Brazil.; 5 Universidade Federal do Triângulo Mineiro Departamento de Fisioterapia Aplicada Uberaba MG Brazil Universidade Federal do Triângulo Mineiro, Departamento de Fisioterapia Aplicada, Uberaba MG, Brazil.

**Keywords:** Stroke, Benchmarking, Artificial Intelligence, Supervised Machine Learning, Emergency Service, Hospital, Acidente Vascular Cerebral, Benchmarking, Inteligência Artificial, Aprendizado de Máquina Supervisionado, Serviço Hospitalar de Emergência

## Abstract

**Background:**

There is a high demand for stroke patient data in the public health systems of middle and low-income countries.

**Objective:**

To develop a stroke databank for integrating clinical or functional data and benchmarks from stroke patients.

**Methods:**

This was an observational, cross-sectional, prospective study. A tool was developed to collect all clinical data during hospitalizations due to stroke, using an electronic editor of structured forms that was integrated with electronic medical records. Validation of fields in the electronic editor was programmed using a structured query language (SQL). To store the results from SQL, a virtual table was created and programmed to update daily. To develop an interface between the data and user, the Embarcadero Delphi software and the DevExpress component were used to generate the information displayed on the screen. The data were extracted from the fields of the form and also from cross-referencing of other information from the computerized system, including patients who were admitted to the stroke unit.

**Results:**

The database was created and integrated with the hospital electronic system, thus allowing daily data collection. Quality indicators (benchmarks) were created in the database for the system to track and perform decision-making in conjunction with healthcare service managers, which resulted in improved processes and patient care after a stroke. An intelligent portal was created, in which the information referring to the patients was accessible.

**Conclusions:**

Based on semi-automated data collection, it was possible to create a dynamic and optimized Brazilian stroke databank.

## RESUMO

**Antecedentes:**

Há alta demanda de dados de pacientes com acidente vascular cerebral (AVC) nos sistemas de saúde de países de baixa e média renda.

**Objetivo:**

Desenvolver um banco de dados de AVC para integrar dados clínicos ou funcionais e indicadores de qualidade de pacientes com AVC.

**Métodos:**

Estudo observacional, transversal e prospectivo. Foi desenvolvida uma ferramenta para coletar dados clínicos durante as internações por AVC por meio de um editor eletrônico de formulários estruturados integrado ao prontuário eletrônico. A validação dos campos no editor eletrônico foi programada em linguagem de consulta estruturada (SQL). Para armazenar os resultados da SQL, uma tabela virtual foi criada e programada para atualização diária. Para desenvolver interface entre os dados e o usuário, foram utilizados o *software Embarcadero Delphi* e o componente *DevExpress* para gerar informações apresentadas na tela. Os dados foram extraídos dos campos do formulário e também do cruzamento de outras informações do sistema informatizado, incluindo pacientes internados na unidade de AVC.

**Resultados:**

O banco de dados foi criado e integrado ao sistema eletrônico do hospital, permitindo coleta diária de dados. Indicadores de qualidade foram criados no banco de dados para que o sistema acompanhasse e realizasse a tomada de decisão com os gestores dos serviços de saúde, resultando em melhoria no processo e no atendimento ao paciente após AVC. Foi criado um portal inteligente, no qual eram registradas as informações referentes aos pacientes.

**Conclusões:**

Com a coleta de dados semiautomática, foi possível criar um banco de dados de AVC dinâmico e otimizado em unidade de AVC no Brasil.

## INTRODUCTION

Stroke is the second leading cause of death and disability worldwide. It has a significant socioeconomic impact on low- and middle-income countries, thereby affecting public health status^1–3^. In the last 40 years, databank projects have been implemented to provide information on the clinical courses and outcomes of stroke^4–8^.

Implementation of a databank is important for planning and decision-making by healthcare service managers, in order to improve processes and stroke care outcomes^9^. However, integration of these databanks is limited to large centers and clinical trials, thus restricting access to low and middle-income countries^10^. In accordance with the principles of stroke care lines in developing countries, it is necessary for all quality criteria during stroke hospitalization to be registered in electronic medical records, with the aim of establishing new healthcare policies^11^.

Based on these premises, implementation of a computerized database system containing the main clinical indicators of stroke patients has become a reality in developing countries^12,13^. Over recent years, the introduction of artificial intelligence has helped in development of databases that are integrated in networks, with the capacity to identify clinical data with greater agility and security^14,15^.

Therefore, the aim of this study was to develop an interface for accessing stroke patient data and the main benchmarks in acute stroke care, in order to create a semi-automated databank for implementation in low and middle-income countries.

## METHODS

### Study design, setting and participants

This project with the aim of developing a semi-automated stroke databank was conducted at the stroke unit of Faculdade de Medicina de Botucatu, Botucatu, Brazil. This stroke unit has 10 beds, with an average of 30 patients/month, and belongs to a tertiary-level hospital with 684 beds. The hospital has a hospital information system (HIS) that includes electronic patient records containing clinical and treatment information. All patients who were admitted to the stroke unit were included in this project. The study was approved by the institution’s Ethics in Human Research Committee.

### Procedures

To organize the information, the tool was designed to include initial data, a clinical summary of the presentation, hospital course (including complications and infections), investigation/complementary examinations (including computed tomography [CT], electrocardiography, echocardiography, Holter data, carotid and vertebral duplex data, CT angiography, magnetic resonance imaging [MRI] angiography, control CTs or MRIs and transcranial Doppler) and discharge conditions (including etiological diagnosis, medications, discharge with therapy plan, rehabilitation and destination of the patient). The rules for each subgroup were planned so as to validate the completion of certain fields, all of which were mandatory for the service to be completed.

The database interface was developed on the HIS platform, and clinical data were collected based on the following benchmarks for stroke care: 1) prophylaxis for deep venous thrombosis, starting no later than the second day; 2) hospital discharge with antiplatelet therapy for patients with non-cardioembolic stroke; 3) hospital discharge with oral anticoagulation for patients with atrial fibrillation or atrial flutter; 4) use of antiplatelet agents when indicated, starting on the second day of hospitalization; 5) hospital discharge with statins for patients with atherothrombotic stroke; 6) hospital discharge with prophylactic therapy and rehabilitation plan; 7) percentage of patients with acute cerebrovascular disease; 8) length of hospital stay; 9) complications; 10) stroke type-specific ICD-10; 11) hospital mortality; 12) time to CT <25 min; and 13) door-to-needle time ≤60 min.

### Execution, monitoring and control phase

To execute the development of the tool, an electronic editor of structured forms was used, which was integrated with the electronic medical record platform used in the institution. Parameterized fields and validation rules were created for essential fields. The rules were created in the field editor. Radio button components were used for information with only one choice and checkbox components were used when the information consisted of multiple choices. In addition, the validation of fields was programmed using a structured language, i.e. in structured query language (SQL)^16^.

Mandatory fields were configured in the editor, and each field was parameterized. All patients who were admitted to the stroke unit were included in the database, as this was mandatory at the time of transfer to another ward or at the time of the patient’s discharge or death.

### Databank storage

To store the results from SQL, a virtual table was created and programmed to update daily at 04:00 h. The interface between the data and user was developed using the program Embarcadero Delphi, and the DevExpress component was used to generate the information displayed on the screen.

### Data quality

The stored data were analyzed to check the data quality in accordance with the pre-established rules for creating the tool as mandatory fields with relationships between the fields. A rule was also created to identify patients who were admitted to the stroke unit and then transferred to another unit, but for whom no discharge summary was filled out at any time during hospitalization. In preparing SQL and business rules for both data analysis and for out-of-base patients, the PL/SQL (Procedural Language for SQL) tool was used via Embarcadero. The data were extracted from the fields of the form and also from cross-referencing of other information from the computerized system, such as patients who were admitted to the stroke unit.

## RESULTS

Once extracted, the data were stored in a database, and any type of information could be consulted at any time, thus allowing cross-referencing between the data of the tool and the patients’ demographic profile. The indicators that were established according to the area and database were available in almost real time, with daily updates at 04:00 h. The database began registering patients in August 2018 and has included data from approximately 1,000 stroke patients to date.

Both qualitative and quantitative results can be obtained from the detailed data included in the database. The results can be grouped to create filters that can be applied to any column within the database. This provides an overview of the total number of patients for each parameter, thus resulting in development of appropriate public health policies.

**Figure 1:**
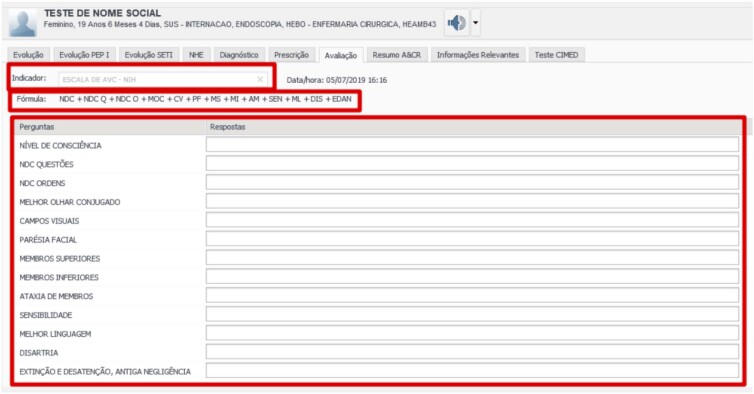
National Institutes of Health Stroke Scale displayed in the electronic medical record.

In addition to the specific data characteristics of stroke patients, and owing to the integration with the electronic medical record, important clinical data such as hospital course, laboratory test results, imaging tests and surgeries, as well as the main scales used in the stroke unit, can be displayed on the screen (Figure 1).

**Figure 2:**
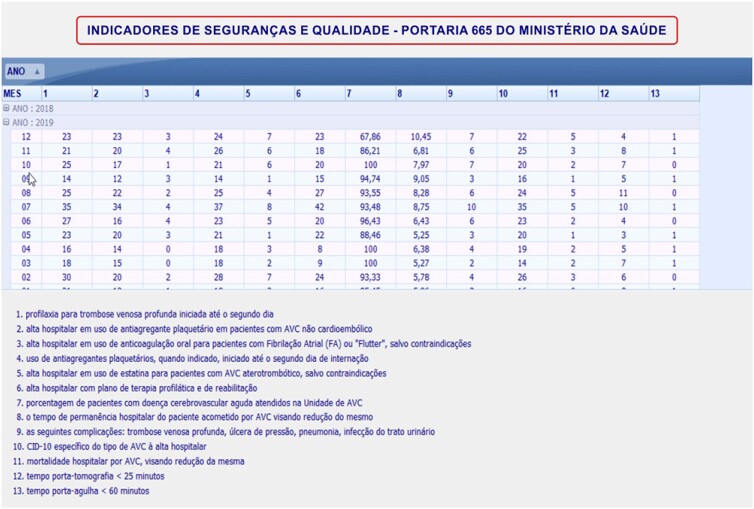
Safety and quality indicators.

The quality indicators (benchmarks) were created in the database for the system to track and perform decision-making in conjunction with healthcare service managers. This resulted in improved processes and patient care after a stroke (Figure 2).

**Figure 3:**
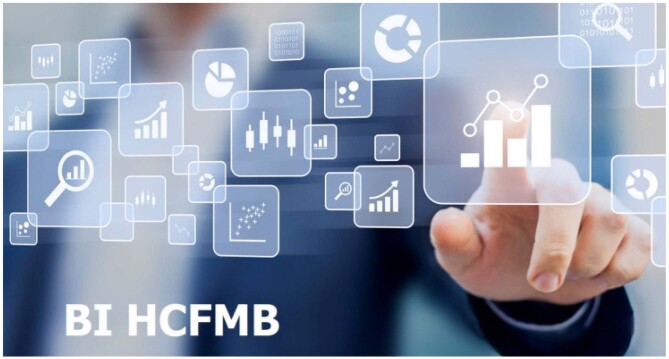
Business intelligence applied to stroke databank.

An intelligent portal was created, in which all the information referring to patients’ care was available in a single location in a dynamic and objective manner. Development of an interface with a specific database for a hospital area enabled creation of a new concept in relation to the data that were already included in the computerized system, thus allowing new ideas to emerge in order to improve and facilitate the interpretation of large volumes of data (Figure 3).

## DISCUSSION

This study reports the development of a tool integrated with electronic medical records that is capable of recording clinical data on patients admitted to a stroke unit and identifying the main indicators of quality of care, using artificial intelligence to improve the quality of care and decision-making process.

Several studies using previous databases have been conducted in low and middle-income countries to identify the quality of healthcare services^17–20^. Zetola et al. observed the registration of patients in a database with identification, previous clinical history, family history, treatments, previous comorbidities, complementary laboratory tests, cardiac tests and neuroimaging tests. Using a computerized system, these data could be extracted dynamically, and their study suggested that a risk factor control program might lead to a reduction in stroke incidence^17^.

In 2013, a single database for stroke patients was established in Joinville, Brazil, via a municipal law that required all public and private healthcare establishments to forward monthly information on stroke patients to the Department of Health to create an integrated database. In 2014, the Ministry of Health coordinated a simultaneous study in five cities in Brazil to assess the incidence of, mortality due to and main risk factors for stroke. The study included individuals with stroke and determined the environmental context of stroke, using a national database of stroke patients in Latin America^21,22^.

The *Rede Brasil AVC* (Brazil Stroke Network), created to improve overall care for stroke patients in Brazil, is a non-governmental organization formed by professionals from various areas with the aim of providing quality care to stroke patients, from prevention to rehabilitation^23^. However, population studies to monitor clinical data on stroke patients need to be integrated into networks with shared access and periodic audits. The development of the tool in this study will help facilitate exploration of clinical data and quality indicators in low and middle-income countries.

Studies on the development of systems containing specific data on stroke patients have not been previously reported. Data collection methods were not reported in previous studies that included databases. Access to information has become increasingly faster and easier through technological advances that enable implementation of structured and standardized electronic databases that use artificial intelligence. Analysis on large datasets of patient characteristics, outcomes from treatments and their costs can help identify the most clinically effective and cost-efficient treatments for a population. Models, analytics, visualization large amounts of data and use of artificial intelligence come together to offer different perspectives on healthcare challenges within the contexts of time and geography. They provide strategic solutions to assist in management, decision-making and future clinical research^24,25^.

An integrated system significantly integrates separate sectors, and teamwork is recorded. This enables top management to fully analyze healthcare processes. Development of this database for stroke patients will optimize this search time and provide large quantities of relevant information for the area available, which can be used to support future studies^26,27^.

In conclusion, through development of integration of this tool and electronic medical records, a dynamic and optimized stroke data bank was created. This database will be useful for managing quality indicators, assisting in the planning of actions at the stroke unit and supporting decision-making, and will serve as a basis for future studies and generation of new knowledge.

## References

[1] Johnston SC, Mendis S, Mathers CD (2009). Global variation in stroke burden and mortality: estimates from monitoring, surveillance, and modelling. Lancet Neurol.

[2] Grysiewicz RA, Thomas K, Pandey DK (2008). Epidemiology of ischemic and hemorrhagic stroke: incidence, prevalence, mortality, and risk factors. Neurol Clin.

[3] Feigin VL, Lawes CM, Bennett DA, Barker-Collo SL, Parag V (2009). Worldwide stroke incidence and early case fatality reported in 56 population-based studies: a systematic review. Lancet Neurol.

[4] Bronstein K, Murray P, Licata-Gehr E, Banko MA, Kelly-Hayes M, Fast S (1986). The Stroke Data Bank project: implications for nursing research. J Neurosci Nurs.

[5] Kunitz SC, Gross CR, Heyman A, Kase CS, Mohr JP, Price TR (1984). The Pilot stroke data bank: definition, design, and data. Stroke.

[6] Foulkes MA, Wolf PA, Price TR, Mohr JP, Hier DB (1988). The Stroke Data Bank: design, methods, and baseline characteristics. Stroke.

[7] Ahmed N, Wahlgren N, Grond M, Hennerici M, Lees KR, Mikulik R (2010). Implementation and outcome of thrombolysis with alteplase 3–4.5 h after an acute stroke: an updated analysis from SITS-ISTR. Lancet Neurol.

[8] Wahlgren N, Ahmed N, Davalos A, Ford GA, Grond M, Hacke W (2007). Thrombolysis with alteplase for acute ischaemic stroke in the Safe Implementation of Thrombolysis in Stroke-Monitoring Study (SITS-MOST): an observational study. Lancet.

[9] Hinchey JA, Shephard T, Tonn ST, Ruthazer R, Selker HP, Kent DM (2008). Benchmarks and determinants of adherence to stroke performance measures. Stroke.

[10] Rossaneis MA, Gabriel CS, Haddad MCFL, Melo MRAC, Bernardes A (2014). Knowledge on health indicators by nurses in hospitalization unities. Rev Eletr Enf.

[11] Cavalcante RB, Cunha SGS, Bernardes MFVG, Gontijo TL, Guimarães EAA, Oliveira VC (2012). Hospital Information System: use in decision making. Journal of Health Informatics.

[12] Patrício CM, Maia MM, Machiavelli JL, Navaes AN (2011). O prontuário eletrônico do paciente no sistema de saúde brasileiro: uma realidade para os médicos. Sci Med.

[13] Brazil - Ministério da Saúde Portaria nº665 (2012). https://bvsms.saude.gov.br/bvs/saudelegis/gm/2012/PRT0665_12_04_2012.html.

[14] Nemati HR, Steiger DM, Iyer LS, Herschel RT (2002). Knowledge warehouse: an architectural integration of knowledge management, decision support, artificial intelligence and data warehousing. Decis Support Syst.

[15] Uraikul VU, Chan CW, Tontiwachwuthikul P (2007). Artificial intelligence for monitoring and supervisory control of process systems. Eng Appl Artif Intell.

[16] Project Management Institute Learn about PMI.

[17] Project Management Institute (2017). Guia do Conhecimento em Gerenciamento de Projetos. Guia PMBOK.

[18] Araújo CC, Oliveira CA, Cruz C, Daniel D, Souza J, Caserta M Gerenciamento de Banco de Dados: Análise Comparativa de SGBD’S.

[19] Rolim CLRC, Martins M (2011). Quality of care for ischemic stroke in the Brazilian Unified National Health System. Cad Saúde Pública.

[20] Rolim CLRC, Martins M (2012). Computerized tomography utilization for stroke inpatients in the Brazilian Health System. Rev Bras Epidemiol.

[21] Araújo JP, Darcis JVV, Tomas ACV, Mello WA (2018). Mortality trend due to cerebrovascular accident in the City of Maringá, Paraná between the years of 2005 to 2015. Int J Cardiovasc Sci.

[22] Zétola VHF, Nóvak EM, Camargo CHF, Júnior HC, Coral P, Muzzio JA (2001). Acidente vascular cerebral em pacientes jovens: análise de 164 casos. Arq Neuro-Psiquiatr.

[23] Martins SCO, Sacks C, Hacke W, Brainin M, Figueiredo FA, Pontes OM (2019). Priorities to reduce the burden of stroke in Latin American countries. Lancet Neurol.

[24] Cabral NL, Freire AT, Conforto AB, Dos Santos N, Reis FI, Nagel V (2017). Increase of stroke incidence in young adults in a middle-income country: a 10-year population-based study. Stroke.

[25] Rede Brasil AVC Rede Brasil AVC.

[26] Raghupathi W, Raghupathi V (2014). Big data analytics in healthcare: promise and potential. Health Inf Sci Syst.

[27] Wang L, Alexander CA (2016). Stroke care and the role of big data in healthcare and stroke. Rehabil Sci.

